# Impact of Cataract Surgery on Postural Control and Fall Risk in Adults and the Elderly: A Systematic Review

**DOI:** 10.3390/medsci14060654

**Published:** 2026-10-09

**Authors:** Juliano Pereira, Rodrigo Franco de Oliveira, Tamyris Mendes de Faria Sudré, Matheus André R. da Costa, Bruna A. Pires Franco de Oliveira, Amélia de Oliveira Pereira, Gabriel de Oliveira Pereira, Luis V. F. Oliveira, Carlos Hassel Mendes da Silva, Deise Aparecida de A. P. Oliveira, Diego Galace Freitas

**Affiliations:** 1 Graduate Program in Health Sciences, Faculty of Medical Sciences of Santa Casa de São Paulo (PPGCS), São Paulo 01224-001, SP, Brazil; juliano@hoa.med.br (J.P.); oliveira.lvf@gmail.com (L.V.F.O.); carloshmendes@unievangelica.edu.br (C.H.M.d.S.); diegogalace@yahoo.com.br (D.G.F.); 2 Anápolis Ophthalmology Hospital (AOH), Anápolis 75080-410, GO, Brazil; amelia@hoa.med.br (A.d.O.P.); gabriel@hoa.med.br (G.d.O.P.); 3 Human Movement and Rehabilitation Graduate Program, Evangelical University of Goiás (UniEVANGÉLICA), Anápolis 75083-515, GO, Brazil; deisepyres@gmail.com; 4 School of Physiotherapy, Evangelical University of Goiás (UniEVANGÉLICA), Anápolis 75083-515, GO, Brazil; tamyrismendesf@hotmail.com (T.M.d.F.S.); matheus1401ribeiro@gmail.com (M.A.R.d.C.); 5 Campus Goianésia, University of Rio Verde (UNIRV), Rio Verde 75901-970, GO, Brazil; bruna.apfranco@gmail.com; 6 Campus Taguatinga, Catholic University of Brasília (UCB), Brasília 71966-700, DF, Brazil

**Keywords:** cataract surgery, postural balance, falls, visual function, older adults

## Abstract

**Background**: Cataracts are a major cause of reversible visual impairment worldwide, particularly among older adults. Visual deterioration associated with cataracts may compromise spatial perception, postural control, and balance, thereby increasing the risk of falls. In this context, cataract surgery has emerged not only as an effective intervention for visual rehabilitation but also as a potential strategy to improve mobility and functional performance in aging populations. **Objective**: The objective of this study was to systematically evaluate the impact of cataract surgery on postural control, balance, and fall risk in adults and older individuals by comparing preoperative and postoperative outcomes. **Methods**: A systematic review was conducted following a protocol registered in PROSPERO. Searches were initially conducted in PubMed, Web of Science, Cochrane Library, and Scopus on 20 October 2025, and rerun on 10 March 2026, which was considered the final search date. Twelve studies were included, encompassing prospective cohorts, retrospective population-based analyses, and comparative studies. The certainty of evidence was assessed using the GRADE framework, and the methodological quality of individual studies was evaluated using JBI critical checklists. **Results**: The included studies generally reported improvements in visual function following cataract surgery, while several studies also reported favorable changes in postural stability, functional balance, and fall-related outcomes. However, findings varied according to study design, outcome definition, surgical timing, and follow-up period. Some studies reported more favorable fall-related and postural outcomes after second-eye surgery; however, the observational nature of the evidence and heterogeneity across studies preclude determining whether bilateral surgery itself caused these improvements. **Conclusions**: Cataract surgery appears to be associated with improved visual and postural function and, in several studies, with reduced fall incidence, particularly after bilateral correction. However, due to substantial heterogeneity, the predominance of observational evidence, and discordant findings on severe injurious outcomes, the certainty of this effect remains limited. The procedure should be regarded as a component of visual rehabilitation that may coincide with reduced fall risk in selected contexts, rather than a universal prevention strategy. Further prospective studies with standardized outcomes are needed to clarify the relationship between cataract surgery, visual function, postural control, and fall-related outcomes.

## 1. Introduction

Cataracts remain the leading cause of reversible visual impairment worldwide, with a high prevalence among adults and older adults attributable to natural aging [1]. The associated loss of visual acuity significantly compromises postural control and increases fall risk, an event of major public health concern in geriatrics due to its substantial clinical and socioeconomic consequences, including fractures, functional decline, and increased mortality [2].

Previous systematic reviews have reported an association between cataract extraction and changes in fall incidence, particularly following bilateral surgery. However, these earlier syntheses present notable limitations. They have predominantly relied on fall counts as the primary outcome, often without distinguishing between minor self-reported events and severe injuries requiring hospitalization. Furthermore, most prior reviews did not systematically integrate objective measures of postural control, functional balance, or visual functions beyond acuity, such as contrast sensitivity and stereopsis, despite their recognized mechanistic relevance to postural stability. The differential impact of first-eye versus second-eye surgery also remains insufficiently clarified in existing syntheses [3,4].

Consequently, several clinically relevant questions remain unanswered. It is unclear whether the observed reduction in self-reported falls after first-eye surgery is accompanied by parallel improvements in instrumented balance, or whether transient increases in severe fall-related injuries reported in some population-based studies represent a consistent phenomenon. The specific contribution of restored stereopsis and contrast sensitivity to postural stability, as well as the optimal timing of second-eye surgery to minimize inter-surgical anisometropia-related risk, have not been adequately addressed in previous review-level evidence [5].

Since the publication of earlier systematic reviews, new primary studies, including prospective cohorts with instrumented balance assessments and large retrospective population-based analyses, have expanded the evidence base. These more recent investigations have introduced nuanced findings, such as the distinction between short-term risk elevation during the inter-surgical interval and long-term protective effects after bilateral completion, as well as data on specific intraocular lens modalities and their influence on functional outcomes. The present review was therefore designed to move beyond the scope of previous syntheses by explicitly incorporating postural control, functional balance, and fall risk as interconnected outcomes. By distinguishing between unilateral and bilateral surgery, between self-reported and severe injurious falls, and between short-term and long-term postoperative periods, this updated review provides a more detailed clinical perspective. Its specific contribution lies in providing clinicians and health policymakers with a nuanced understanding of the contexts and conditions in which cataract surgery may be associated with changes in postural control and fall-related outcomes, thereby supporting more individualized surgical planning and postoperative care [6].

Thus, this study aims to systematically analyze the impact of cataract surgery on postural control, balance, and fall risk in adults and older adults, comparing preoperative and postoperative periods. A comprehensive understanding of these relationships is essential to guide clinical practice and inform care strategies that promote health and quality of life in this population.

## 2. Methods

This study consists of a systematic review without meta-analysis, aimed at analyzing the impact of cataract extraction surgery on postural control, balance, and fall risk in adults and older adults. Given the substantial clinical and methodological heterogeneity of the included studies, particularly regarding outcome definitions, assessment instruments, and follow-up periods, a quantitative synthesis was not deemed appropriate. Instead, a narrative synthesis was performed to integrate the evidence qualitatively. The decision not to conduct a meta-analysis was based on differences in study designs, surgical timing, comparator conditions, outcome definitions, assessment instruments, follow-up periods, and effect measures. These differences also remained within individual outcome domains, limiting the feasibility of pooling studies even when they addressed similar outcomes. Therefore, the findings were synthesized narratively according to outcome type, surgical timing, comparison group, and study design [7].

The research question was structured according to the PICO framework. The included population (P) comprised adults and older adults with cataracts who underwent cataract surgery. Cataract extraction refers to the surgical removal of the opacified lens. Phacoemulsification refers to a cataract extraction technique in which the lens nucleus is fragmented using ultrasonic energy and subsequently aspirated, whereas phacoextraction is a broader term referring to surgical removal of the crystalline lens using an extraction technique. The intervention (I) therefore included studies involving cataract extraction procedures, particularly phacoemulsification, with or without intraocular lens implantation, according to the procedures described in the original studies. For the comparison (C), eligible studies included preoperative versus postoperative comparisons, operated versus non-operated groups, comparisons between first-eye and second-eye surgery, and other comparative designs reported within the eligible observational studies. The outcomes (O) evaluated included postural control, balance, and fall-related outcomes [8].

The primary outcomes were postural control, functional balance, and falls. Falls were considered according to the definitions adopted by each included study, including self-reported falls, fall incidence, and fall-related injuries requiring hospitalization. Postural and functional balance outcomes included validated clinical or instrumented measures reported by the individual studies. Visual function outcomes, including visual acuity, contrast sensitivity, and stereopsis, were also extracted when reported because of their potential relationship with postural control and falls.

The review protocol was prospectively registered in PROSPERO (CRD420251149332) and is publicly accessible through the PROSPERO database at https://www.crd.york.ac.uk/PROSPERO/view/CRD420251149332, accessed on 17 September 2025.

### 2.1. Search Strategy

The search strategy was designed to ensure full conceptual alignment with the PICO question and the eligibility criteria. Three conceptual domains were defined: (i) cataract and surgical extraction; (ii) postural balance, postural control, and related constructs; and (iii) accidental falls, fall risk, and related events. Each domain was populated with controlled vocabulary (DeCS/MeSH) and free-text terms, combined with the Boolean operator OR within domains and AND between domains.

The narrative synthesis was structured according to study design, population characteristics, surgical timing, outcome definition, assessment instruments, and follow-up period.

The electronic searches were conducted in PubMed, Web of Science, Cochrane Library, and Scopus. All databases were initially searched on 20 October 2025. Because the search strategies were subsequently modified, all databases were rerun on 10 March 2026, and this date was considered the final search date for the present review. The search strings were substantially modified, specifically the inclusion of the balance/postural control domain in PubMed and Cochrane. The complete search strategies for each database, including controlled vocabulary, free-text terms, Boolean operators, and database-specific syntax, are presented in Table 1.

### 2.2. Study Selection

Study selection was performed independently by two reviewers in two stages. First, titles and abstracts were screened according to the predefined eligibility criteria. Subsequently, potentially eligible studies were assessed in full text. The screening process was conducted using Rayyan© v1.7.8 [9]. Disagreements between the two reviewers were resolved through discussion and, when consensus was not reached, by consultation with a third reviewer. The same procedure was applied during the full-text assessment. Reviewers were blinded to each other’s decisions during the initial screening process.

Studies involving adult and elderly patients undergoing cataract surgery that included an assessment of postural control, balance, and/or fall risk, published in Portuguese, English, or Spanish, without restriction on the year of publication, were included. Original observational and comparative studies evaluating adults or older adults undergoing cataract surgery were eligible for inclusion. No statistical transformation or conversion of effect estimates was performed because the review used a narrative synthesis without meta-analysis. Data were extracted as reported in the original studies and organized into standardized descriptive tables.

Studies involving pediatric populations, those that did not address cataract surgery as the main intervention, or those that did not include assessment of postural control, balance, or fall risk were excluded. Abstracts, letters to the editor, editorials, literature reviews, and duplicate studies were also excluded.

### 2.3. Data Extraction

Data extraction was performed independently by two reviewers using a standardized data extraction form. The extracted information included author, year of publication, study design, sample size, population characteristics, type of cataract surgery, assessment instruments, follow-up period, and outcomes related to postural control, balance, and falls. Any disagreements between reviewers were resolved through discussion and, when necessary, consultation with a third reviewer.

Because this review did not perform a meta-analysis, effect measures were reported according to those used in the individual studies. These included risk ratios (RR), incidence rate ratios (IRR), hazard ratios (HR), adjusted hazard ratios (aHR), odds ratios (OR), changes in continuous balance measures, and corresponding 95% confidence intervals when available.

### 2.4. Risk of Bias Assessment

The methodological quality of the included studies was assessed using the Joanna Briggs Institute (JBI) critical appraisal checklists, version 2020 [10]. For cohort studies, the 11-item checklist was used; for case–control studies, the 10-item checklist; and for cross-sectional/prospective observational study, the 8-item checklist. Each item was classified as “Yes,” “No,” “Not applicable,” or “Not reported.” Studies meeting ≥ 70% of the applicable criteria were considered to have low risk of bias. The assessment was performed independently by two reviewers, with disagreements resolved by consensus or, when necessary, by arbitration by a third reviewer (Table 2).

The certainty of the evidence for each outcome was assessed using the Grading of Recommendations Assessment, Development and Evaluation (GRADE) approach [11]. The certainty of evidence was classified as high, moderate, low, or very low based on methodological limitations, inconsistency, indirectness, imprecision, and publication bias. Cataract surgery has been extensively investigated regarding its effects on the risk of falls, postural balance, and visual acuity in adults and elderly patients. Prospective observational study and retrospective studies indicate that cataract surgery, especially when performed on both eyes, has heterogeneous associations with fall-related and postural outcomes. Because no meta-analysis or quantitative synthesis was performed, a formal statistical assessment of publication bias or small-study effects was not conducted. Potential concerns regarding selective outcome reporting were considered qualitatively during the interpretation of the included studies.

**Table 2:** Assessment of the risk of bias of the included studies according to the JBI checklist.

Study	Type of Design	1	2	3	4	5	6	7	8	9	10	11	Score	%
Study 1 [12]	Longitudinal cohort	●	●	●	●	●	●	●	●	●	●	●	11/11	100
Study 2 [13]	Retrospective cohort	●	●	●	●	●	●	●	●	○	●	●	10/11	90.9
Study 3 [14]	Retrospective cohort	●	●	●	●	●	●	●	●	●	●	●	11/11	100
Study 4 [15]	Prospective observational study	●	●	●	●	●	●	●	●	—	—	—	8/8	100
Study 5 [16]	Case–control	●	●	●	●	●	●	●	○	●	●	—	9/10	90
Study 6 [17]	Prospective cross-sectional	●	●	●	●	●	●	●	●	—	—	—	8/8	100
Study 7 [18]	Longitudinal cohort	●	●	●	●	●	●	●	●	●	●	●	11/11	100
Study 8 [19]	Retrospective cohort	●	●	●	●	●	●	●	●	●	●	●	11/11	100
Study 9 [20]	Retrospective cohort	●	●	●	●	●	●	●	●	●	●	●	11/11	100
Study 10 [21]	Analytical cross-sectional study	●	●	●	●	●	●	●	●	—	—	—	8/8	100
Study 11 [22]	Retrospective cohort	●	●	●	●	●	●	●	●	●	●	●	11/11	100
Study 12 [23]	Longitudinal cohort	●	●	●	●	●	●	●	●	●	●	●	11/11	100

**Source**: Produced by the authors, 2026. ● = yes; ○ = no; — = not applicable.

Cataracts remain the leading cause of reversible visual impairment in the world, with high prevalence among adults and older adults, due to natural aging [1].

## 3. Results

After the selection of eligible studies, the extraction and organization of the main methodological information and outcomes analyzed in each research were carried out. Aspects such as authorship, year of publication, study design, sample characteristics, type of surgical intervention, and main results related to postural control, balance, and fall risk were considered. The synthesis of this information is systematized in Table 3, which presents the data extraction from the studies included in this review.

**Figure 1:**
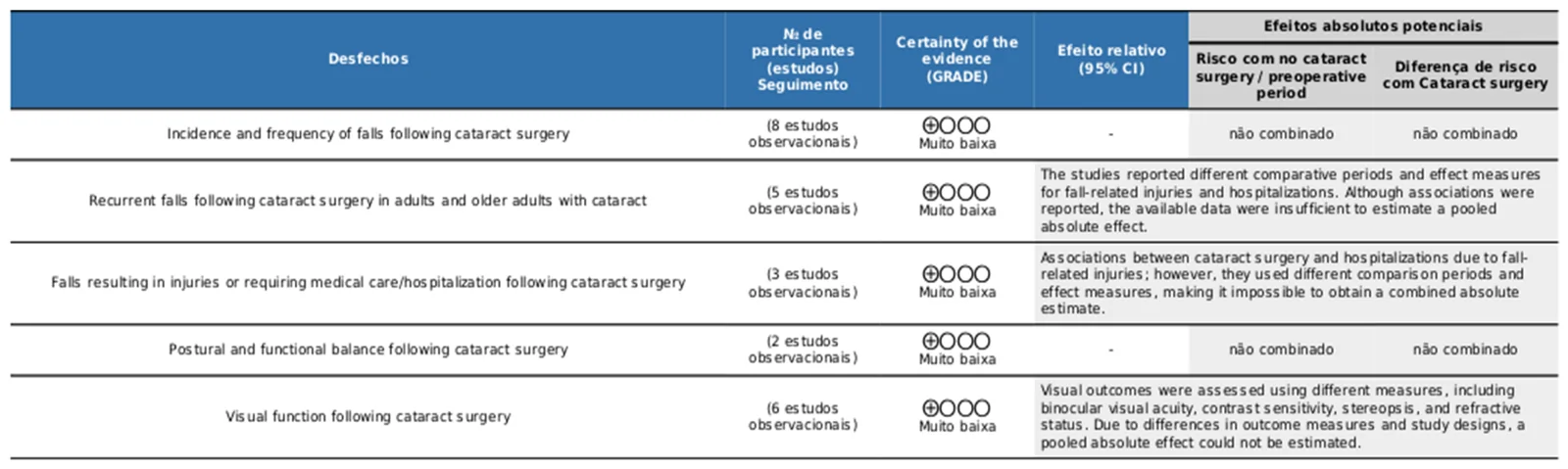
Certainty of Evidence—GRADE. **++++** = High certainty. **+++-** (or +++) = Moderate certainty. **++--** (or ++) = Low certainty. **+---** (or +) = Very low certainty.

The certainty of evidence for the evaluated outcomes was assessed using GRADE and is presented in Figure 1. The certainty was downgraded when applicable because of methodological limitations, inconsistency, indirectness, imprecision, or concerns regarding publication bias.

The systematic review included twelve studies that investigated the relationship between cataract surgery and the risk of falls in the elderly. Among these, six prospective studies evaluated cohorts with follow-up periods ranging from six months to two years, four retrospective population-based studies analyzed Australian administrative databases with over 28,000 individuals, and two comparative studies examined types of intraocular lenses. The prospective and comparative studies employed objective assessments of visual function and balance, while the population-based studies focused on severe outcomes such as hospitalizations for fractures and intracranial hemorrhages.

### 3.1. First-Eye Surgery: Divergent Effects by Outcome Definition and Study Design

Due to substantial clinical and methodological heterogeneity among the included studies regarding population characteristics, surgical procedures, outcome definitions, assessment instruments, and follow-up periods, quantitative pooling was not performed because the studies differed substantially in outcome definitions, surgical timing, comparator groups, assessment methods, follow-up periods, and effect measures. Combining these estimates would therefore require assumptions that were not supported by the available evidence. Therefore, a narrative synthesis was performed. Studies were grouped according to the main outcomes evaluated, including falls, postural control and functional balance, and visual function, and findings were compared according to surgical timing, first- versus second-eye surgery, follow-up period, and study design. Prospective clinical cohorts evaluating self-reported falls have demonstrated significant protective effects, with risk reductions ranging from 54% to 78% in the postoperative period [12,18]. Brannan et al. (2003) [15], in a clinical study with 97 patients, observed a marked reduction in the proportion of individuals who suffered falls, from 37% preoperatively to 7% postoperatively, reinforcing the benefit of the intervention for self-reported events.

Conversely, retrospective population-based analyses of Australian administrative databases have reported divergent findings for severe injurious outcomes. These studies documented a 27% increase in hospitalizations due to fall-related injuries during the first postoperative year and a doubled risk of injurious falls in the interval between first- and second-eye surgeries [13,20]. This discrepancy is not methodologically contradictory but rather reflects distinct outcome definitions and observation contexts. Minor self-reported falls, commonly captured in prospective clinical studies, differ fundamentally from severe events requiring hospitalization, which are tracked in administrative data. Furthermore, increased medical surveillance in the immediate postoperative period may enhance detection and reporting of severe events. A critical transient factor is inter-surgical anisometropia, which reaches a mean refractive difference of 2.68 diopters after the first procedure, affecting four out of five patients [16], potentially compromising depth perception and postural stability during the inter-surgical interval.

### 3.2. Bilateral Surgery and Long-Term Outcomes: Consolidation of Protection

The completion of second-eye surgery appears to consolidate the visual and functional benefits, with the apparent increased risk observed during the first-eye and inter-surgical periods being transient. Following bilateral correction, prospective cohorts have documented sustained risk reductions of 73% to 83% compared to the preoperative period [12,23], with the annual incidence of self-reported falls decreasing from 1.17 to 0.41 events per patient [23]. Similar favorable associations have also been reported for some severe fall-related outcomes, with population-based studies indicating reductions in hip fractures, proximal humerus and distal radius fractures, as well as subdural and epidural intracranial hemorrhages after bilateral completion [19].

Direct comparative studies reinforce the importance of binocular visual symmetry. Pseudophakic patients with bilateral correction exhibit significantly superior binocular visual acuity compared to those with untreated bilateral cataracts (mean difference of 0.72 logMAR) and a lower incidence of falls, regardless of systemic comorbidities [16]. Unilateral correction provides partial visual rehabilitation; however, these findings suggest that binocular visual function may be relevant to postural and fall-related outcomes, although the available evidence does not establish that complete binocular symmetry is necessary for fall prevention.

### 3.3. Visual Mechanisms and Functional Balance

The relationship between visual improvement and fall reduction transcends simple visual acuity. Contrast sensitivity and stereopsis show a more consistent association with the risk of falls than acuity measured on the Snellen chart [17]. Instrumented balance assessments, such as the Berg and Tinetti scales, record significant improvements after surgery, especially in patients with previously more compromised vision [21]. The magnitude of the improvement in balance does not correlate linearly with the gain in visual acuity, indicating the involvement of complex sensory-motor integration mechanisms.

### 3.4. Risk Factors and Characteristics of the Intraocular Lens

Patients with a history of falls in the previous 12 months constitute a subgroup of heightened vulnerability, with a 2.5-fold higher risk during the surgical waiting period [18]. Regarding the type of intraocular lens, the low prevalence of multifocal implants limits the robustness of this conclusion [20]. Pseudophakic monovision does not alter the risk of falls, while pseudophakic single vision may transiently increase it [22]. The late opacification of hydrophilic lenses in surgery combined with endothelial keratoplasty highlights the importance of monitoring optical quality in complex procedures [17].

Sex-related differences were also reported in some of the included studies. Fraser et al. [12] observed a higher incidence of falls among women, with women presenting approximately three times the risk observed in men (IRR 3.13). In the longitudinal cohort by Keay et al. [23], women represented 54% of the sample. Rosenblatt et al. [22] also identified female sex as a risk factor in the non-operated group. However, these findings were not consistently evaluated across all included studies, and sex-specific analyses were not sufficiently homogeneous to determine whether sex modifies the effect of cataract surgery on fall risk. Therefore, the observed differences should be interpreted as potential risk characteristics rather than as evidence of a differential treatment effect.

An additional consideration concerns the use of multifocal or bifocal spectacles. Although the included study evaluating multifocal intraocular lenses did not demonstrate a statistically significant increase in fall-related hospitalizations compared with monofocal lenses, evidence from outside the present surgical literature indicates that multifocal spectacle use may influence fall risk under specific circumstances. The VISIBLE randomized controlled trial reported that providing single-lens distance glasses to older multifocal spectacle wearers reduced falls in certain outdoor environments, particularly among individuals with previous falls. These findings suggest that the optical demands associated with different forms of multifocal correction should be distinguished from the effects of multifocal intraocular lenses. Therefore, the available evidence remains insufficient to determine whether specific intraocular lens modalities independently modify fall risk after cataract surgery.

**Figure 2:**
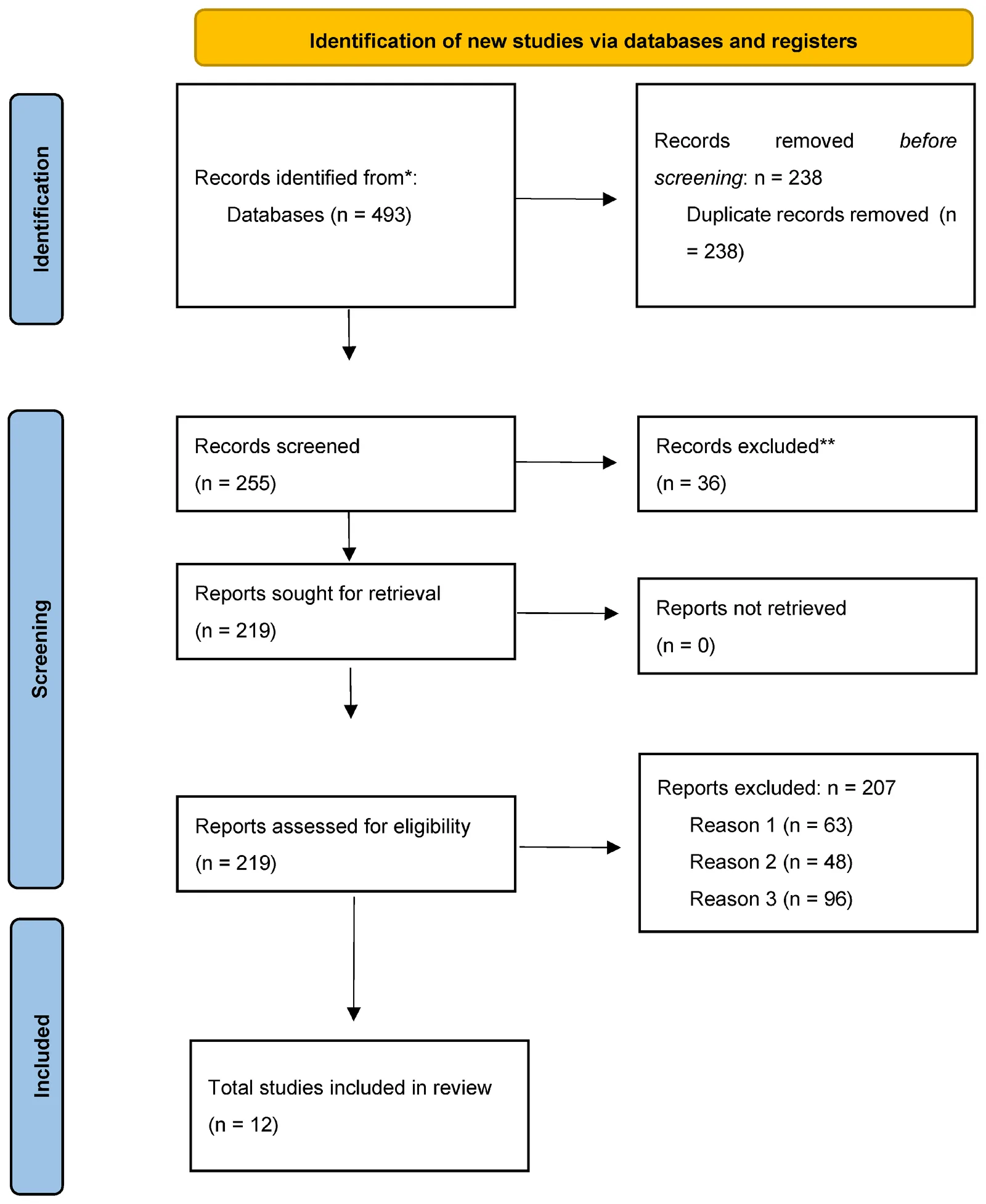
PRISMA 2020 flow diagram of the study selection process. Note: Reason 1: absence of intervention with cataract surgery; Reason 2: non-evaluation of outcomes of interest; Reason 3: insufficient data for outcome analysis. * Records identified from databases. ** Records excluded during title and abstract screening. Source: Adapted by the authors, 2026.

The process of identification, screening, eligibility, and inclusion of studies was conducted according to methodological recommendations for systematic reviews. Initially, the identified records were evaluated for duplication and subsequently subjected to screening through the reading of titles and abstracts. The potentially eligible studies were analyzed in full to verify the established inclusion criteria. The details of the selection stages, as well as the number of studies included and excluded at each phase, are presented in the PRISMA flowchart (Figure 2), while the PRISMA checklist is presented in Table 4 [24].

Across the 12 included studies, sample sizes varied substantially, ranging from clinical cohorts with fewer than 100 participants to population-based studies involving thousands of individuals. The included populations comprised adults and older adults, with variation in age distribution, sex, geographic setting, and clinical characteristics. Because several studies were based on large administrative or population databases, the reported sample sizes should not be interpreted as representing a single pooled population, and potential overlap between study populations could not be excluded from the information available in the included reports. Detailed sample characteristics, follow-up periods, surgical timing, and comparison groups are presented in Table 3.

## 4. Discussion

The present systematic review indicates that cataract surgery is associated with improvements in visual function and, in several studies, with favorable changes in postural balance and fall-related outcomes among adults and older adults. However, the magnitude and direction of these associations varied according to outcome definition, surgical timing, study design, and follow-up period. These findings reinforce the central role of vision in the sensorimotor integration necessary for postural stability, especially in aging populations. Cataracts progressively compromise visual acuity, contrast sensitivity, and depth perception, impairing spatial orientation and obstacle detection, which directly impacts gait and balance [25].

In this context, falls constitute an important public health problem, associated with high morbidity, mortality, and loss of functional independence.

It is estimated that about one-third of individuals over 65 experience at least one fall per year, with visual impairment being an independent risk factor due to its influence on the integration between the visual, vestibular, and somatosensory systems [26,27].

The present systematic review indicates that cataract surgery is associated with improved visual function and, in several studies, with favorable changes in postural balance and fall-related outcomes in adults and older adults [25]. An important consideration is that the potential effect on falls may be mediated primarily by the restoration of visual function rather than by the surgical procedure itself [15]. Cataract surgery improves visual acuity, contrast sensitivity, and, particularly after bilateral correction, binocular visual function, which may contribute to better spatial orientation, depth perception, and postural control. Therefore, the relationship between cataract surgery and falls should be interpreted within the broader pathway linking visual impairment, restoration of vision, and functional mobility rather than as a direct causal effect of surgery alone [1].

However, a clinically relevant finding is the existence of a transitional period of increased vulnerability between the surgery of the first and second eye. Evidence indicates a temporary increase in the risk of falls and hospitalizations during this interval; a potential mechanism that may contribute to this transitional vulnerability is inter-surgical anisometropia. The refractive imbalance between the operated and non-operated eyes may temporarily affect binocular visual integration, depth perception, and visuomotor coordination. However, this mechanism remains a plausible explanation rather than an established causal pathway, as the included studies did not consistently directly measure anisometropia and its relationship with falls [14,23]. This phenomenon highlights the importance of proper surgical planning, including the timely performance of the second eye surgery and refractive correction in the intermediate period.

From a methodological standpoint, the heterogeneity among the included studies should be considered in the interpretation of the results. Differences in study design, prospective versus retrospective, in fall measurement methods, self-reported versus hospitalizations, in follow-up periods, and in population characteristics can influence the magnitude of the observed effects. Additionally, confounding factors such as comorbidities, physical activity level, and environmental conditions can independently impact the outcomes [26,28]. The predominance of observational studies also implies greater susceptibility to confounding and selection biases, limiting the inference of direct causality.

Despite the methodological limitations, the findings indicate an association between restoration of visual function after cataract surgery and favorable changes in balance and fall-related outcomes in some populations. However, the observational nature and heterogeneity of the available evidence do not allow cataract surgery to be interpreted as an independent causal intervention for fall prevention. In summary, cataract surgery transcends visual rehabilitation, emerging as an intervention with potential impact on fall prevention and the preservation of functionality in the elderly. The incorporation of strategies such as preoperative assessment of fall risk, monitoring during the intersurgical period, and timely planning of bilateral approach can optimize the observed clinical and functional outcomes.

Statistical significance should be distinguished from clinical relevance when interpreting the observed changes in balance and functional outcomes. Although some studies reported statistically significant postoperative improvements, the clinical importance of these changes depends on the magnitude of the improvement, the variability of the measurement, and the availability of established minimal clinically important differences for the instrument used. Because the included studies used different assessment tools and did not consistently report standardized effect sizes or clinically meaningful thresholds, the extent to which statistically significant changes translate into clinically important improvements in everyday mobility remains uncertain.

## 5. Conclusions

The findings of this systematic review indicate that cataract surgery is consistently associated with improved visual function and, in some studies, with improvements in postural control, functional balance, and fall-related outcomes. However, the direction and magnitude of these associations varied according to study design, outcome definition, surgical timing, and follow-up period. The predominance of observational studies, methodological heterogeneity, and low or very low certainty of evidence limit causal inference regarding the effect of cataract surgery on fall prevention. Therefore, cataract surgery should be considered primarily as a visual rehabilitation intervention that may also be associated with favorable functional and fall-related outcomes in selected populations. Further prospective studies using standardized measures of postural control, balance, and falls are needed to clarify these relationships.

However, the certainty and magnitude of this effect remain heterogeneous across the included studies. The evidence base is predominantly observational, and the results are not uniform: while prospective clinical cohorts report reductions in self-reported falls after both first- and second-eye surgeries, population-based retrospective analyses have identified transient increases in severe fall-related injuries requiring hospitalization during the first postoperative year and in the inter-surgical interval. One possible explanation is transient visual imbalance during the inter-surgical period, potentially related to refractive differences between the operated and unoperated eyes; however, this mechanism was not directly demonstrated in the included studies.

Therefore, cataract surgery may be considered an important component of visual rehabilitation and may be associated with improvements in balance and fall-related outcomes in some populations, particularly following bilateral correction. Nevertheless, the substantial clinical and methodological heterogeneity and the predominance of observational evidence prevent definitive conclusions regarding a causal effect of cataract surgery on fall prevention. Future prospective studies using standardized outcome measures and clearly defined follow-up periods are needed to clarify the relationship between visual restoration, postural control, and fall-related outcomes.

## Figures and Tables

**Table 1:** Descriptors used in the databases.

Database	Descriptors
PubMed	(“Cataract Extraction”[Mesh] OR “cataract surgery”[Title/Abstract] OR “cataract extraction”[Title/Abstract] OR “phacoemulsification”[Title/Abstract]) AND (“Postural Balance”[Mesh] OR “postural control”[Title/Abstract] OR balance[Title/Abstract] OR “postural stability”[Title/Abstract] OR “functional balance”[Title/Abstract]) AND (“Accidental Falls”[Mesh] OR fall[Title/Abstract] OR “fall risk”[Title/Abstract] OR “fall incidence”[Title/Abstract])
Web Of Science	TS = (“Cataract Surgery” OR “Cataract Extraction” OR “Phacoemulsification”) AND TS = (“Postural Control” OR “Postural Stability” OR “Postural Equilibrium” OR “Postural Balance”) AND TS = (“Accidental Falls” OR “Fall Risk” OR “Risk of Falling”)
Cochrane Library	(“Cataract Extraction”: ti, ab, kw OR “Cataract Surgery”: ti, ab, kw OR “Phacoemulsification”: ti, ab, kw) AND (“Postural Control”: ti, ab, kw OR “Postural Stability”: ti, ab, kw OR “Postural Equilibrium”: ti, ab, kw OR “Postural Balance”: ti, ab, kw OR “Balance Control”: ti, ab, kw) AND (“Accidental Falls”: ti, ab, kw OR “Fall Risk”: ti, ab, kw OR “Risk of Falling”: ti, ab, kw)
Scopus	TITLE-ABS-KEY( (“cataract surgery” OR “cataract extraction”) AND (“postural control” OR “postural stability”) AND (“fall risk” OR “risk of falling”)

Source: Produced by the authors, 2026.

**Table 3:** Data extraction.

Study	Title	Objective	Methodology	Main Results	Conclusion
Study 1 [12]	A longitudinal cohort study of the impact of first- and both-eye cataract surgery on falls and other injuries in Vietnam	Determine the impact of cataract surgery on the first eye and both eyes on the number of falls and other injuries in patients with bilateral cataracts in Ho Chi Minh City, Vietnam.	Longitudinal cohort with 413 patients aged ≥50 years and bilateral cataract. Assessments: 1 week pre-surgery, 1–3 months, and 1 year after the first eye surgery. Visual acuity, contrast sensitivity, stereopsis, and self-reported falls/injuries were measured. Analysis by longitudinal multilevel Poisson regression.	The surgery of the first eye reduced the risk of falls by 78% (IRR 0.22; 95% CI 0.06–0.77), and the surgery of the second eye by 83% (IRR 0.17; 95% CI 0.04–0.69). Women had a three times higher risk than men (IRR 3.13). The improvement in binocular contrast sensitivity correlated with a lower incidence of falls, as well as a reduction in the prevalence of other injuries.	The study found an association with lower the number of falls and other injuries. Contrast sensitivity can be an important factor in surgical prioritization and fall risk assessment.
Study 2 [13]	The impact of first- and second-eye cataract surgery on injurious falls that require hospitalisation: a whole-population study	Evaluate the risk of fall-related injury in adults aged ≥60 years in the 2 years before the first eye surgery, between the first and second eye surgeries, and in the 2 years after the second eye surgery.	Retrospective cohort with 28,396 individuals aged ≥60 years who underwent bilateral cataract surgery in Western Australia (2001–2008). Sources: Western Australian Hospital Morbidity Data System and Death Registry. Analysis using Poisson regression with generalized estimating equations, adjusted for confounders.	The risk of fall with hospitalizable injury doubled between surgeries (RR 2.14; 95% CI 1.82–2.51) compared to the 2 years preoperatively. In the 2 years following the second surgery, there was a 34% increase in these falls (RR 1.34; 95% CI 1.16–1.55) compared to the baseline period.	An increase in the risk of falls with injury was observed after the surgery of the first and second eye, highlighting the importance of timely surgery of the second eye and appropriate refractive management during the interval between procedures.
Study 3 [14]	First eye cataract surgery and hospitalization from injuries due to a fall: a population-based study	Evaluate the association between cataract surgery in the first eye and hospitalizations due to fall-related injuries in adults aged ≥60 years at the population level.	Retrospective population-based study with 15,295 individuals aged ≥60 years who underwent unilateral cataract surgery (2004–2008). Sources: Western Australian Hospital Morbidity Data System and mortality records. Outcome: Hospitalization for fall-related injuries 1 year before and 1 year after surgery. Analysis by Poisson regression with generalized estimating equations.	Among the 15,295 individuals who underwent surgery, 600 (3.9%) had 625 hospitalizations due to fall-related injuries. There were 27% more hospitalizations in the year following the surgery compared to the previous year (adjusted RR = 1.27; 95% CI 1.04–1.56; *p* = 0.02). About 30% of the hospitalizations were due to femoral neck fractures.	Cataract surgery on the first eye was associated with an increase in hospitalizations due to fall-related injuries in the year following the procedure. More studies are needed to clarify the factors involved.
Study 4 [15]	A prospective observational study of the rate of falls before and after cataract surgery	Verify if cataract surgery reduces the risk of falls in elderly patients with age-related cataracts.	Prospective clinical study with 97 patients scheduled for cataract surgery. Evaluation of risk factors for falls in the preoperative and postoperative periods. Recording of falls in a diary and bimonthly phone follow-up for 6 months before and after the surgery. Final sample: 84 patients.	In the preoperative period, 31 patients (37%) reported falls. In the postoperative period, there was a significant reduction in the number of patients who experienced falls (n = 6; *p* < 0.001).	Cataract surgery was associated with a significant reduction in the risk of falls in elderly patients with cataract-related visual impairment.
Study 5 [16]	Impact of first eye cataract surgery on falls among patients of advanced age: a comparative study	Compare the incidence of falls between patients with visually significant bilateral cataracts and patients who underwent first-eye surgery.	Retrospective case–control study with 100 patients (50 in the pseudophakic group—first eye surgery performed between 9 and 12 months; 50 in the bilateral cataract group). Corrected binocular visual acuity (BCVA), systemic comorbidities (Charlson Index), medication use, and activities of daily living (Lawton Scale) were evaluated. The occurrence of ≥2 falls in the last 6 months was investigated.	Binocular visual acuity was significantly better in the pseudophakic group (0.05 ± 0.06 logMAR) compared to the cataract group (0.77 ± 0.34 logMAR). In total, 22% of participants reported ≥2 falls in the last 6 months. Multivariate analysis showed better BCVA in participants with fewer than two falls.	Older adult individuals with bilateral cataracts are at a higher risk of falls. The surgery of the first eye can reduce the occurrence of falls by improving binocular visual acuity.
Study 6 [17]	The Effect of Cataract Surgery on Stereoacuity, Balance, and Falls in Patients With Senile Cataract	Investigate the effect of cataract surgery on visual acuity, stereopsis, balance, and falls in patients with senile cataract.	Prospective cross-sectional study with patients divided according to initial surgery in the dominant eye (Group 1) or non-dominant eye (Group 2). Assessments: complete ophthalmological examination, stereopsis tests (Titmus and TNO), Berg Balance Scale, and falls in the last 36 months. Moments: Pre-operative, 6 months after the first surgery, and 24 months after the second.	The average number of falls decreased from 4.95 ± 2.6/year in the pre-operative phase to 1.43 ± 0.59/year in the post-operative phase, with a 53.15% reduction after the second surgery. There was a moderate negative correlation between preoperative stereopsis and falls (r = −0.546; *p* = 0.033). Low corrected visual acuity in the dominant eye was associated with worse stereopsis and more falls in the preoperative period.	Cataract surgery improves visual acuity, increases stereopsis, enhances balance, and reduces the number of falls. Multicenter studies with larger samples are recommended.
Study 7 [18]	The impact of first and second eye cataract surgeries on falls: a prospective cohort study	Investigate the impact of cataract surgery on the first and second eye on the risk of falls in individuals with bilateral cataracts and identify which changes in visual measures are associated with the change in the number of falls throughout the surgical process.	Longitudinal study with 55 adults aged ≥55 years with bilateral cataract. Evaluations conducted at three time points (pre-first surgery, between first and second surgeries, and post-second surgery). Participants filled out a fall diary. Generalized estimating equations (GEE) models with negative binomial regression were used to analyze the number of falls and their association with visual variables.	After adjusting for confounding factors, there was a 54% reduction in the risk of falls after the first surgery (IRR = 0.458; *p* = 0.04) and a 73% reduction after the second surgery (IRR = 0.268; *p* = 0.002), compared to the pre-first surgery period. Better binocular visual acuity (*p* = 0.029) and better contrast sensitivity (*p* = 0.040) were associated with a reduction in the number of falls.	The surgeries of the first and second eye significantly reduced the risk of falls in elderly patients with bilateral cataracts. The improvement in binocular visual acuity and contrast sensitivity seems to play an important role in this reduction. The timely performance of surgeries can help reduce the burden of falls in this population.
Study 8 [19]	Age-Related Cataract Extraction Is Associated With Decreased Falls, Fractures, and Intracranial Hemorrhages in Older Adults	Investigate the association between cataract surgery with intraocular lens implantation (CEIOL) and the occurrence of falls and other aging-related traumatic injuries, as well as hip fractures.	Retrospective cohort with data from TriNetX. Included patients aged ≥60 years with age-related cataracts, divided into Group 1 (underwent phacoemulsification within 10 years of diagnosis) and Group 2 (not operated on). Matching by propensity score for demographics and comorbidities. Analysis by chi-square and odds ratio (95% CI).	Patients who underwent CEIOL had a lower chance of falls (*p* < 0.0001), proximal humerus fracture (*p* = 0.016), distal radius fracture (*p* = 0.0004), hip fracture (*p* < 0.0001), ankle fracture (*p* = 0.0002), subdural hemorrhage (*p* < 0.0001), and epidural hemorrhage (*p* = 0.006), compared to those who did not undergo surgery.	CEIOL significantly reduced falls and severe traumatic injuries in elderly patients with cataracts, reinforcing the importance of timely screening and treatment as a preventive strategy.
Study 9 [20]	Risk of falls and injuries requiring hospitalisation after first-eye cataract surgery in older adults Australians	Investigate whether the type of intraocular lens (monofocal or multifocal) implanted in the first cataract surgery influences the risk of hospitalization due to falls and injuries in the elderly patients.	Retrospective cohort with data from the Australian Department of Veterans Affairs. Included individuals aged ≥65 years who underwent their first cataract surgery (January 2007–July 2017), stratified by lens type (monofocal or multifocal). Analysis of the risk of hospitalization due to falls and injuries in the first 3 postoperative months using a Cox proportional hazards model, adjusted for age, sex, comorbidities, and previous fall history.	Total of 45,728 participants (97% monofocal lens; 3% multifocal lens). A lower risk of hospitalization due to falls and injuries was observed in the multifocal group (aHR = 0.56; 95% CI 0.26–1.17), but without statistical significance. Age stratification maintained non-significant results.	Multifocal lenses were not associated with a higher risk of hospitalization due to falls and injuries after the first eye surgery, regardless of age.
Study 10 [21]	Impact of Cataract Surgery on Functional Balance Skills of Adults	Investigate the impact of phacoemulsification surgery with intraocular lens implantation on functional balance skills in adults with cataracts.	Prospective study (May–October/2016) with 51 patients (mean age 66.96 years). Application of the Berg Balance Scale and the Tinetti Test in the preoperative period and 1 month after surgery. Analysis according to age, visual acuity, and balance performance, with comparison between patients with worse and better visual acuity.	One month after the surgery, there was an average increase of 3.60 ± 5.00% in the Berg Scale and 4.14 ± 6.55% in the Tinetti Test. The improvement in visual acuity was significantly greater in patients with worse preoperative vision (*p* = 0.036). However, the difference in balance scores between the acuity groups was not statistically significant.	Cataract surgery promotes a significant improvement in visual acuity and an increase in functional balance scores, especially in individuals with poorer preoperative vision, contributing to safer mobility and better quality of life.
Study 11 [22]	Fall risk in patients with pseudophakic monovision	Evaluate the risk of falls in elderly patients with bilateral cataracts undergoing pseudophakic monovision, pseudophakic single vision, or no cataract surgery.	Single-institution retrospective cohort of 13,385 patients aged ≥60 years with bilateral cataracts, excluding those with unilateral surgery or previous falls. Source: Stanford Research Repository. Main outcome: Hazard ratio (HR) of fall after second surgery or bilateral diagnosis.	Of the 13,385 patients, 850 experienced falls. After adjustment, pseudophakic monovision was not associated with a higher risk. On the other hand, simple pseudophakic vision reduced the time until the first fall compared to the non-operated (*p* < 0.001). Advanced age, female sex, and pre-existing myopia increased the risk in the non-operated.	Pseudophakic monovision does not alter the risk of falls, while surgery with a single vision lens may increase the risk of falls compared to patients without cataract surgery.
Study 12 [23]	The incidence of falls after first and second eye cataract surgery: a longitudinal cohort study	Compare the incidence of falls, visual acuity, and refractive status before surgery, after the first cataract surgery, and after the second cataract surgery.	Prospective observational study in eight ophthalmology referral clinics in public hospitals in Sydney, Melbourne, and Perth. Included patients aged ≥65 years with bilateral cataracts between 2013–2016, followed for up to 24 months or up to 6 months after the second surgery. Primary outcome: Incidence of falls adjusted for age and sex. Secondary outcomes: Visual acuity and refractive error.	There were 409 participants (mean age 75.4 years; 54% women). Adjusted incidence of falls: 1.17/year in the preoperative period, 0.81/year after the first eye, and 0.41/year after the second. In the 118 patients with complete follow-up, the incidence was 0.80/year before, 0.81/year after the first surgery, and 0.32/year after the second. Average binocular visual acuity (logMAR): 0.32 preoperatively, 0.15 after the first eye, and 0.07 after the second.	The surgery on the first eye significantly improves vision, but the second surgery is necessary to effectively reduce the incidence of falls. Timely bilateral surgery optimizes vision and reduces the risk of fall-related injuries in the elderly.

**Source**: Produced by the authors, 2026. Cataract surgery has been extensively investigated regarding its effects on the risk of falls, postural balance, and visual acuity in adults and the elderly. The included prospective and retrospective studies reported heterogeneous associations between cataract surgery and fall-related or postural outcomes, with several studies reporting more favorable outcomes following bilateral surgery.

**Table 4:** PRISMA checklist, 2020.

Section and Topic	Item #	Checklist Item	Location Where Item Is Reported
**TITLE**			
Title	1	Identify the report as a systematic review.	p. 1, Title
**ABSTRACT**			
Abstract	2	See the PRISMA 2020 [24], for Abstracts checklist.	p. 1, Abstract
**INTRODUCTION**			
Rationale	3	Describe the rationale for the review in the context of existing knowledge.	pp. 3–4, Section 1
Objectives	4	Provide an explicit statement of the objective(s) or question(s) the review addresses.	p. 4, Section 1
**METHODS**			
Eligibility criteria	5	Specify the inclusion and exclusion criteria for the review and how studies were grouped for the syntheses.	pp. 4–6, Section 2, Section 2.1 and Section 2.2; eligibility criteria
Information sources	6	Specify all databases, registers, websites, organisations, reference lists and other sources searched or consulted to identify studies. Specify the date when each source was last searched or consulted.	p. 5, Section 2.1; search date and databases reported, but the final rerun date is not specified
Search strategy	7	Present the full search strategies for all databases, registers and websites, including any filters and limits used.	p. 5, Table 1; full database-specific strategies
Selection process	8	Specify the methods used to decide whether a study met the inclusion criteria of the review, including how many reviewers screened each record and each report retrieved, whether they worked independently, and if applicable, details of automation tools used in the process.	pp. 6–7, Section 2.2; Rayyan, two independent reviewers, third-reviewer resolution
Data collection process	9	Specify the methods used to collect data from reports, including how many reviewers collected data from each report, whether they worked independently, any processes for obtaining or confirming data from study investigators, and if applicable, details of automation tools used in the process.	p. 7, Section 2.3; two independent reviewers and third-reviewer resolution
Data items	10a	List and define all outcomes for which data were sought. Specify whether all results that were compatible with each outcome domain in each study were sought (e.g., for all measures, time points, analyses), and if not, the methods used to decide which results to collect.	pp. 4, 6, Section 2 and Section 2.3
	10b	List and define all other variables for which data were sought (e.g., participant and intervention characteristics, funding sources). Describe any assumptions made about any missing or unclear information.	p. 6, Section 2.3
Study risk of bias assessment	11	Specify the methods used to assess risk of bias in the included studies, including details of the tool(s) used, how many reviewers assessed each study and whether they worked independently, and if applicable, details of automation tools used in the process.	p. 7, Section 2.3; participant/intervention variables reported; assumptions for missing/unclear information not explicitly stated
Effect measures	12	Specify for each outcome the effect measure(s) (e.g., risk ratio, mean difference) used in the synthesis or presentation of results.	p. 7, Section 2.3; RR, IRR, HR, aHR, OR, continuous balance measures, 95% CI when available
Synthesis methods	13a	Describe the processes used to decide which studies were eligible for each synthesis (e.g., tabulating the study intervention characteristics and comparing against the planned groups for each synthesis (item #5)).	pp. 9–14, Table 3
	13b	Describe any methods required to prepare the data for presentation or synthesis, such as handling of missing summary statistics, or data conversions.	Not clearly reported
	13c	Describe any methods used to tabulate or visually display results of individual studies and syntheses.	pp. 9–14, Table 3
	13d	Describe any methods used to synthesize results and provide a rationale for the choice(s). If meta-analysis was performed, describe the model(s), method(s) to identify the presence and extent of statistical heterogeneity, and software package(s) used.	pp. 4, 9–19, Section 2, Section 3 and Section 4
	13e	Describe any methods used to explore possible causes of heterogeneity among study results (e.g., subgroup analysis, meta-regression).	Not reported
	13f	Describe any sensitivity analyses conducted to assess robustness of the synthesized results.	Not reported
Reporting bias assessment	14	Describe any methods used to assess risk of bias due to missing results in a synthesis (arising from reporting biases).	p. 7, Section 2.3; data extracted as reported and no statistical conversion; handling of missing summary statistics not explicitly detailed
Certainty assessment	15	Describe any methods used to assess certainty (or confidence) in the body of evidence for an outcome.	p. 7, Section 2.3; standardized descriptive tables
**RESULTS**			
Study selection	16a	Describe the results of the search and selection process, from the number of records identified in the search to the number of studies included in the review, ideally using a flow diagram.	pp. 16–17, Figure 2
	16b	Cite studies that might appear to meet the inclusion criteria, but which were excluded, and explain why they were excluded.	Not reported
Study characteristics	17	Cite each included study and present its characteristics.	p. 5, Section 2.1; p. 15, Section 3; qualitative heterogeneity explored by design, population, surgical timing, outcome definition, instruments and follow-up
Risk of bias in studies	18	Present assessments of risk of bias for each included study.	N/A—no sensitivity analysis was performed because no meta-analysis was conducted
Results of individual studies	19	For all outcomes, present, for each study: (a) summary statistics for each group (where appropriate) and (b) an effect estimate and its precision (e.g., confidence/credible interval), ideally using structured tables or plots.	p. 7, Section 2.4; formal statistical assessment of publication bias not conducted; selective outcome reporting considered qualitatively
Results of syntheses	20a	For each synthesis, briefly summarise the characteristics and risk of bias among contributing studies.	pp. 15–19, Section 3 and Section 4
	20b	Present results of all statistical syntheses conducted. If meta-analysis was done, present for each the summary estimate and its precision (e.g., confidence/credible interval) and measures of statistical heterogeneity. If comparing groups, describe the direction of the effect.	N/A—no meta-analysis was performed
	20c	Present results of all investigations of possible causes of heterogeneity among study results.	N/A—no formal heterogeneity analysis was performed
	20d	Present results of all sensitivity analyses conducted to assess the robustness of the synthesized results.	N/A—no sensitivity analysis was performed
Reporting biases	21	Present assessments of risk of bias due to missing results (arising from reporting biases) for each synthesis assessed.	p. 18, Figure 2; PRISMA flow diagram
Certainty of evidence	22	Present assessments of certainty (or confidence) in the body of evidence for each outcome assessed.	Not reported—potentially eligible full-text studies excluded are not individually cited with reasons
**DISCUSSION**			
Discussion	23a	Provide a general interpretation of the results in the context of other evidence.	pp. 18–19, Section 4
	23b	Discuss any limitations of the evidence included in the review.	pp. 18–19, Section 4
	23c	Discuss any limitations of the review processes used.	p. 18, Section 4
	23d	Discuss implications of the results for practice, policy, and future research.	pp. 18–19, Section 4 and Section 5
**OTHER INFORMATION**			
Registration and protocol	24a	Provide registration information for the review, including register name and registration number, or state that the review was not registered.	p. 4, Section 2
	24b	Indicate where the review protocol can be accessed, or state that a protocol was not prepared.	Not reported
	24c	Describe and explain any amendments to information provided at registration or in the protocol.	Not reported
Support	25	Describe sources of financial or non-financial support for the review, and the role of the funders or sponsors in the review.	pp. 15–20, Table 3 and Section 3; effect estimates and 95% CI when available
Competing interests	26	Declare any competing interests of review authors.	pp. 15–20, Section 3; narrative synthesis and contributing study characteristics/risk of bias
Availability of data, code and other materials	27	Report which of the following are publicly available and where they can be found: template data collection forms; data extracted from included studies; data used for all analyses; analytic code; any other materials used in the review.	N/A—no meta-analysis/statistical synthesis was performed

**Note**: **#** N/A, not applicable; not reported, information not reported in the manuscript.

## Data Availability

No new data were created or analyzed in this study. Data sharing is not applicable to this article.
